# Development of a Low-Cost Electronic Nose for Detection of Pathogenic Fungi and Applying It to *Fusarium oxysporum* and *Rhizoctonia solani*

**DOI:** 10.3390/s21175868

**Published:** 2021-08-31

**Authors:** Piotr Borowik, Leszek Adamowicz, Rafał Tarakowski, Przemysław Wacławik, Tomasz Oszako, Sławomir Ślusarski, Miłosz Tkaczyk

**Affiliations:** 1 Faculty of Physics, Warsaw University of Technology, ul. Koszykowa 75, 00-662 Warszawa, Poland; pborow@poczta.onet.pl (P.B.); Rafal.Tarakowski@pw.edu.pl (R.T.); Przemyslaw.Waclawik@pw.edu.pl (P.W.); 2 Forest Protection Department, Forest Research Institute, ul. Braci Leśnej 3, 05-090 Sękocin Stary, Poland; T.Oszako@ibles.waw.pl (T.O.); S.Slusarski@ibles.waw.pl (S.Ś.); M.Tkaczyk@ibles.waw.pl (M.T.)

**Keywords:** electronic nose, odour classification, VOC, volatile organic compounds, fungi and biosecurity

## Abstract

Electronic noses can be applied as a rapid, cost-effective option for several applications. This paper presents the results of measurements of samples of two pathogenic fungi, *Fusarium oxysporum* and *Rhizoctonia solani*, performed using two constructions of a low-cost electronic nose. The first electronic nose used six non-specific Figaro Inc. metal oxide gas sensors. The second one used ten sensors from only two models (TGS 2602 and TGS 2603) operating at different heater voltages. Sets of features describing the shapes of the measurement curves of the sensors’ responses when exposed to the odours were extracted. Machine learning classification models using the logistic regression method were created. We demonstrated the possibility of applying the low-cost electronic nose data to differentiate between the two studied species of fungi with acceptable accuracy. Improved classification performance could be obtained, mainly for measurements using TGS 2603 sensors operating at different voltage conditions.

## 1. Introduction

Analysis and detection of odours can be performed using many techniques of chemical analysis of gases. On the one hand, most of the information can be obtained by classical chemical analytical techniques, such as gas chromatography combined with mass spectrophotomety [1]. It allows identifying the individual chemical components of a sample and their relative concentrations. Unfortunately, in practice, this technique is limited to applications in laboratory conditions. On the other hand, there are multiple approaches for which so-called electronic nose devices (e-nose) have been proposed [2,3,4]. They do not identify the components of a gas mixture, but rely on the pattern recognition techniques supported by machine learning algorithms. E-noses are usually rapid, non-invasive, online instruments comprised of an array of gas sensors and appropriate pattern recognition software.

Different types of gas sensors can be used in the development of electronic noses, such as optical [5], gravimetric [6] and electrochemical [7] sensors. Specifically, low-cost simple constructions are often based on commercially available metal-oxide (MOX) sensors. In the recent years, several groups reported such devices based on Taguchi-type MQ series gas sensors [8,9,10,11,12,13,14]. Other devices were proposed by Szczurek et al. [15] to detect bee colony infestations, Wu et al. [16] for cigarette brand identification, Anyfantis and Bliona [17] to detect human presence, and Gonzalez Viejo et al. [18] to assess the aroma profiles of beer. Lampson and coworkers have described a portable electronic nose for detecting pests and plant damage [19].

An essential task in the process of electronic nose construction is a choice of gas sensor types. Multiple reports describe approaches to optimisation of the gas sensor arrays [20,21,22,23,24,25]. Several approaches allow even single sensor devices to be used, wherein different aspects of the measurement conditions are investigated to achieve the required sensitivity and selectivity. The most often used approach is to use all characteristics of the sensor’s response to the changes in gas condition from clean air to the sample (gas adsorption), and again to the clear air (gas desorption), which exploits transient sensors’ response regions [26,27,28,29,30]. Another commonly used approach is to exploit the sensor’s response in the regime modulated sensor temperature [31,32,33,34,35].

When building a low-cost electronic nose, the constructors are limited in their choices of gas sensors to the models on the market. Therefore, the standard approach is to use electrical circuit parameters according to recommendations of the sensor manufacturers—for example, the sensor’s heater voltage, which determines the sensor’s temperature. On the other hand, since the MOX gas sensors are used for other purposes in electronic noses than originally intended, different operation conditions could be more appropriate. Fonollosa and coworkers [36] reported research on optimisation of MOX sensor array temperature. There is also research on MOX [37] and nanomaterial [38] sensor arrays, during which multiple sensors of the same type were working in different temperatures.

Proposed constructions, applications, or limitations and challenges of e-noses designed for studies of fungal, bacterial, and viral infections can be found in several review papers [39,40,41,42,43]. Electronic noses have also found applications in cheese ripening analysis [44,45]. These devices have been used to confirm the originality of, e.g., Italian Parmigiano Reggiano, and its quality (ripening time). The portable S3 sensing device, based on a matrix of six metal oxide semiconductor gas sensors, proved to be fast and reliable, and the SPME-GC-MS method allowed the identification of biomarkers of volatile organic compounds (VOC); the results were evaluated using multivariate statistical principal component analysis (PCA). Mota et al. (2021) [46] examined published information on the feasibility of fungal identification using electronic noses. The results of 16 articles showed that a system based on electronic sensors can detect mycotoxins and identify the associated microbial species. This technology has already been tested in several domains, from the food industry to clinical practice.

Herein, we report experimental results of a simple, low-cost electronic nose device. When in a sensor array, we used two sensors and five individual sensors working in different temperatures. The device was successfully used to differentiate between odours of two common forest fungi pathogens, *Fusarium oxysporum* and *Rhizoctonia solani*. Fungi of the genus *Fusarium*, belonging to Ascomycota, and *Rhizoctonia*, belonging to Basidiomycota, have been of interest to microbiologists worldwide, for many years. Currently, these fungi are counted among the most pathogenic and phytotoxic microorganisms in the world. They are plant pathogenic fungi with broad host ranges and worldwide distribution. The species *R. solani* was discovered over 100 years ago and is considered a soilborne pathogen, and *F. oxysporum* is currently considered a species complex comprising a variety of species and strains that are ubiquitous in soils [47]. Most of these strains are saprotrophic, and despite their ability to colonise plant roots, some are represented as commensal endophytes that do not affect their host plants [48].

The most recent review paper, still referred to by many researchers, dates back more than 35 years [49]. The authors reported 79 particular forms and mentioned races in 16 unique forms. Since then, the known host range of *F. oxysporum* has expanded considerably, and many new particular forms and races have been described. In an extensive literature review, Edel-Hermann and Lecomte [50] enumerated 106 special forms that they considered well documented, 37 special forms that they considered insufficiently documented, and 58 additional host plants for which no special form has yet been characterised. The researchers’ studies show the power and danger of the formation of new forms of *F. oxysporum*. The difficulty of traditional detection of these fungi in practice is that the anamorph (imperfect, asexual form) does not produce spores, while the teleomorph (sexual, perfect) does not produce fruiting bodies, but only sexual spores, called basidiospores. A device that detects pathogens in the soil of the rhizosphere or at the base of the stems would be precious for early detection of pathogens and planning preventive measures.

Some authors [51] performed in vitro tests on cellulose agar at two relative humidities (75, 100% RH) to distinguish contamination and colonisation by *Aspergillus terreus, A. holandicus*, and *Eurotium chevalieri*. In vitro tests showed that the conductive polymer sensor matrix exhibited different responses to each species at the two moisture levels. Discriminant functional analysis of the data showed discrimination between the control and the fungi. Cluster analysis showed significant separation of the control from each of the paper-damaging fungi. In situ tests on three paper grades showed that the volatile pattern produced by each fungus differed from the patterns of the others and the control when natural substrates were used. The results obtained were better at higher moisture contents. The three paper grades were successfully differentiated into clusters. The differentiation between the control and the perishable fungi was better for a single paper grade with higher moisture content. This study showed that this technology can detect fungal decay in library and archival materials early to protect cultural heritage better. We also want to acknowledge here a paper by Falasconi et al. [52], in which detection by the olfactory system of *Fusarium verticillioides* in corn is reported.

## 2. Electronic Nose

**Figure 1:**
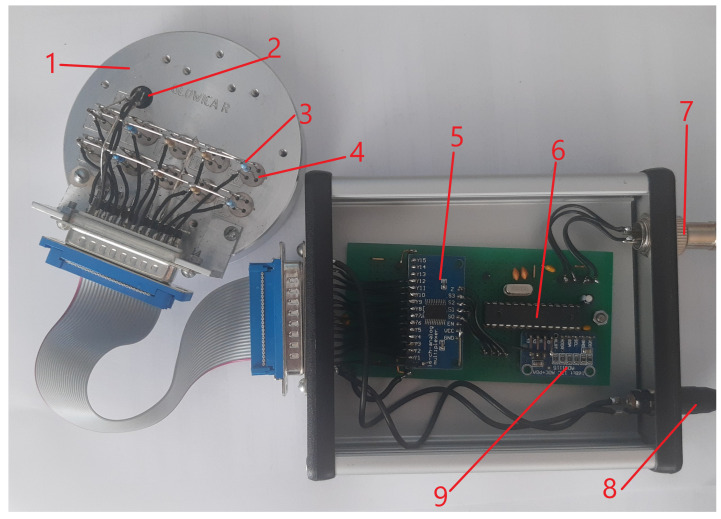
The PW6 electronic nose device. The cover was opened for the photo. 1—aluminium probe, 2—temperature and humidity sensors, 3—additional resistor, 4—TGS sensor, 5—16-channel analogue multiplexer (Texas Instruments, Dallas, TX, USA), 6—Atmel 0814G Atmega 8-16PU microcontroller (Microchip Technology, Chandler, AZ, USA), 7—USB wire, 8—external power supply wire, 9—16-bit ADS1115 AD converter Texas Instruments, Dallas, TX, USA).

In the present paper, we report the results of measurements performed by two electronic nose devices. PW4 was already described in more detail in the previous paper [53], and PW6 is presented in Figure 1.

### 2.1. Sensor Array Selection

The operation of an electronic nose relies on measuring the resistances of non-specific gas sensors exposed to the studied odour sample. In the previous papers [53,54],, we reported the measurements performed by the electronic nose PW4 in which six MOX commercially available gas sensors of Figaro Inc (Japan) were used. The list and target gases of these sensors are presented in Table 1. In previous research, we demonstrated the possibility of differentiating between samples of odours emitted by pathogenic oomycetes *Pythium intermedium* and *Phytophthora plurivora*. We also noticed that the low-cost electronic nose device could be simplified, as the best performing classification was obtained when data extracted from just one sensor signal were used. It was the TGS 2603 sensor, but also, in some cases TGS 2602 sensor data allowed us to achieve similar classification performance. In the current study, we have extended our research to detect odours of pathogenic fungi that are not closely related to oomycetes. Therefore, it can be expected that the emitted metabolites contain similar or different chemical components and yet can be detected by the same type of gas sensors.

Sensor models TGS 2602 and TGS 2603 —the latter especially—are designed to detect low concentrations of odorous gases generated from waste or spoiled materials. Hence, it is not surprising that they react to the gases emitted by the growth of oomycetes or fungi, as this is a similar biological process of organic material consumption as during food spoilage.

When building a low-cost electronic nose based on MOX sensors, we were limited to commercially available sensors, as manufacturing our own sensors would have been too costly. One idea for improving electronic nose performance is to use more sensors with overlapping target gases but with different response characteristics and to then rely on the machine learning algorithms to differentiate between response patterns. For that task, it would be possible to use sensors from various manufacturers. Another idea exploited in this paper is to change the conditions of sensor operation, which can be achieved by changing the sensor’s heater voltage, and thus the operation temperature. This approach is similar to proposals of using an array of custom-made gas sensors with a temperature gradient [37,38].

It may be interesting to recall the construction of an MOX-type gas sensor and its operation principle. The main elements of the sensor are a sensing material, typically tin dioxide, and an electric heater, usually made of platinum. When the sensing material is heated, usually to the temperature of a few hundred degrees Celsius, in clear air, oxygen is absorbed on the surface of the sensing element and attracts donor electrons, preventing electric current flow. In reducing gases, oxygen reacts with the gas particles and the surface density of adsorbed oxygen decreases; donor electrons are released into the tin dioxide, leading to electric current flow. This means that the temperature is an important parameter for controlling the physical processes of this gas sensor’s operating principle.

**Figure 2:**
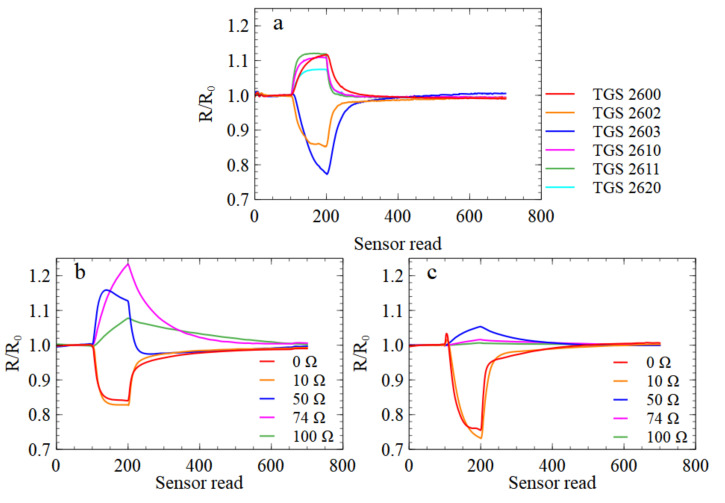
Examples of sensors’ responses to a sample of *Rhizoctonia* odour. Responses of sensors are displayed in the subfigures as marked: (**a**) in PW4 e-nose device, (**b**) in PW6 with TGS2602 sensors and (**c**) with TGS2603 sensors. The y-axis represents the sensor resistance *R* relative to the resistance in clean air $R_{0}$. The x-axis represents the sensor resistance read number counted from the start of the measurement of the sample. The sensors were read for 1.22 seconds each.

To some extent, an MOX gas sensor behaves differently at different temperatures, and may exhibit different sensitivities and selectivities to various chemical components. Indeed, in Figure 2, we present the response curves of sensors of the same type but with various sensor heater voltages, and one can notice that their response characteristics can be significantly different. In addition, even if the odour classification method has the best performance when based on the data collected by a single sensor, studies on the dependence of the electronic nose’s performance on the sensor temperature are essential, as they allow one to determine optimal measurement conditions [36].

### 2.2. Electronic Nose Construction

Compared to PW4, the PW6 device has upgraded hardware: a 16-bit ADS1115 AD converter instead of a 12-bit one. To control more sensors, a better model of the Atmega microchip controller (Atmel 0814G Atmega 8-16PU) was used. Additional delays were added to ensure that the time of one measurement sequence was the same for both devices. As the sensors’ heaters voltage stability was critical in this experiment, an external power supply was used to power them. Sensors were power supplied, just as in PW4, via a computer’s USB port. This cable was also used to control the devices and send the data to the computer, where they were stored.

The PW6 electronic nose device was built using ten metal oxide gas sensors (Figaro co., Osaka, Japan) and humidity and temperature sensors. We decided not to use any air pumps or fans to force airflow to reduce costs and complications. The sensors were placed in an aluminium cover that just fit into Petri dishes 9 cm in diameter. The wires that connect sensors and multiplexers were connected alternately to signal and ground wires, to better shield electrical noise.

**Figure 3:**
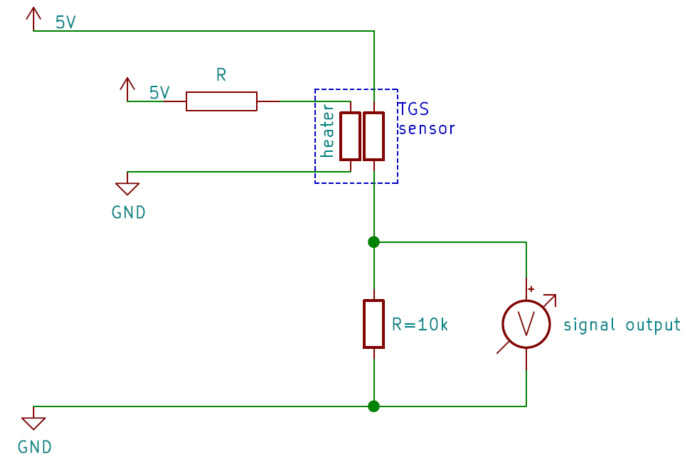
The scheme of wiring for a representative sensor. There was an additional resistor, allowing us to modify the electric voltage applied to the sensor’s heater and thus reduce the sensing element’s temperature.

The main difference in the PW6 e-nose, compared to the PW4, was the use of only two types of sensors, but each of them had an extra resistor R in the sensor’s heater circuit, presented in Figure 3. After the tests reported in the previous papers [53,54], two models of air pollutant sensors, TGS 2602 (sensitive to VOCs, ammonia, and H${}_{2}$S) and TGS 2603 (sensitive to amine and sulphur series odours), were chosen. They were connected to resistors, as listed in Table 2. This combination made ten different sensors in the electronic nose array. This resistor changed the power supplied to the sensors’ heaters by changing the voltages to them. Power gained by the heater was proportional to the square of the voltage and inversely proportional to the resistance. The voltages measured on each heater are presented in Table 2.

Due to the different original resistances of the heaters (TGS 2602 had 59 Ω and TGS 2603 had 67 Ω, both at room temperature), the temperatures of the different types of sensor with the same resistor were not the same. Additionally, the resistances of the heaters changed with their temperatures. The different parameters allowed by different sensor manufacturers cause different sensitivities and reactions from sensors of the same type. As low-coast devices, PW4 and PW6 do not need much power to work. The main power consumers are the sensors’ heaters. The levels of power consumption by individual components of the constructed devices are presented in Table 3.

## 3. Measured Samples

Damping-off is a disease that causes decay of germinating seeds and young seedlings, especially those that grow in forest nurseries [61]. Among a lot of different organisms causing damping-off that were taken for testing, *F. oxysporum* and *R. solani* were chosen for further examination with the use of the constructed e-nose device. They are the most abundant pathogens in Poland’s forest nurseries, and they can create chlamydospores (thick-walled large resting spores). Pathogens in this form can remain latent in the soil for a very long time and develop when optimal development conditions (e.g., weakness in plants and high humidity) occur. It is one of many reasons why their early detection is so essential in nursery production.

The pathogenic strains mentioned above develop readily in soil, which is their natural habitat. Their adaptability has led them to being considered one of the most damaging microorganisms in the world today. They cause root rot [62,63], tuber blight [64], and wilting. In addition, recent evidence indicates that the species *F. oxsyporum* is among the top 10 most destructive fungal plant pathogens worldwide. The pathogenic strains of *F. oxysporum* are responsible for two types of symptoms. The most common is vascular wilt, and in some cases, rotting. In vascular wilt, *Fusarium* invades host roots in the xylem vessels, which it colonises upward, causing progressive yellowing and wilting of the plant. While residing in the soil, it can decompose dead organic matter (saprotrophic), or infest and damage healthy plants (parasite), or live a latent life in their tissues (endophyte). Over time, the endophytic presence of the pathogen may be manifested by reduced plant growth, followed by wilting, chlorosis, and premature death of the plant. This shows how important it is to be able to detect it at an early stage. The fungus *F. oxysporum* is the only representative of the genus *Fusarium* that develops in the vascular system of the host plant and spreads upwards within the plant tissue, mainly affecting the bark layer. Other fungal species spread outside the plant tissue. In the presence of suitable host plants, *Fusarium* changes its mode of life to parasitic (occasional parasite). An infection by a pathogen of the genus *Fusarium* occurs more readily when the root system is mechanically damaged; nematode damage is particularly favoured.

The difficulty of controlling a species as complex as *F. oxysporum* is that there are harmful, toxic, and pathogenic strains and beneficial ones. Some strains of *F. oxysporum*, such as Fo47 and CS-20, are indeed beneficial to the host and may even protect against root pathogens. At this stage, our research should only distinguish between *F. oxysporum* and *R. solani* in order to use effective selective fungicides. An additional consideration is that the *F. oxysporum* species complex mutates frequently, and virulence-related genes may lead to the continual emergence of new races within the species [65,66,67,68].

*R. solani*, in turn, lives in the soil as vegetative hyphae or spores called sclerotia. The thick cell walls of the spores allow them to survive in the soil for many years. They begin to develop when exposed to chemicals secreted by a growing plant, or when exposed to chemicals produced during the decomposition of organic plant debris. Capturing these volatiles with an e-nose is a solution that provides some promise. The fungus can enter the plant through natural openings and through the epidermis. Upon contact with the plant, the cuticle of the pathogen produces appressoria and infectious hyphe that penetrate the plant cells. Enzymes produced by the pathogen are involved in the dissolution of the cuticle.

### 3.1. Sample Preparation

The fungal strains were isolated in a forest nursery (Chojnów Forest District) from the rhizosphere soil of diseased European oaks (*Quercus robur*) and were kept in the stock of the Department of Forest Protection in the Forest Research Institute in Sękocin Stary (Poland). The fungal samples were cultured on classical PDA-Agar media (20 g dextrose, 15 g agar, 4 g potato starch, and 1 L distilled water) in 9 cm Petri dishes. They were kept at room temperature until the mycelium grew over the surfaces of their dishes completely.

### 3.2. Measurements of Samples

The procedure of odour measurements was almost the same as in earlier work [53]. It was detailed in the previous paper, so we outline only the most essential parts here. For both devices, the measuring procedure was the same, and the devices were used simultaneously. Each day of measurements included at least one series of measurements for all samples. The sequence of samples was selected via random number generator separately for each device at the beginning of each day. The number of one-day measurement series was limited to two to avoid overestimation of any day. During any one day, the sensors’ responses to the odour of one type of sample looked similar.

Each measurement included 700 sensor reads; readings were taken over 1.22 s each. The first 100 reads (122 s) were the baseline. Next, the sensors were placed manually on the Petri plate with the sample. That made a small gas chamber under the sensors. After 100 readings, each sensor’s probe was removed and placed away from the samples. The last 500 resistance readings (6 min 10 s) recorded relaxation and sensor cleanings. When not being measured, all samples on Petri dishes were covered to avoid any infections. Electronic noses and samples were stored in a laminar flow cabinet to ensure stable environmental conditions.

All data used in this paper were measured between the 23rd of June and the 7th of July 2021. That makes 10 measuring days, so any random noise in the environment could be reduced. The sensors were power supplied and heated during all those days. We kept them clean. For each of the specimens, 35 measurements were collected. That makes a total of 105 records to analyse.

In our experiment, we maintained controlled conditions of constant temperature and humidity throughout the experiment. The sensitivity of the MOX gas sensors is highly dependent on these parameters, and we were interested in finding differences in the responses of the sensors to the odours emitted by the test samples. Since we held the humidity constant, we could consider a sensor’s response to it as a background signal, which was the same in all cases.

## 4. Data Analysis Techniques

The data analysis that we performed consisted of several steps. First of all, the sensors’ response resistance data, which are presented in Figure 2, were pre-processed, and in this step, several features describing the shapes of the curves were extracted. We applied principal component analysis (PCA) to transform the input dataset to less dimensional space and visualise data distribution patterns. The main type of data analysis involved building machine learning classification models to differentiate between categories. All analyses of experimental data that we report were performed using Python 3.7 code with the scikit-learn module.

### 4.1. Data Preprocessing

The number of reads collected during one measurement of a sample was 3600 for the PW4 e-nose and 6000 for the PW6 e-nose device—each represents the number of sensors multiplied by the number of reads of sensor resistance magnitude in both the gas adsorption and desorption phases, as one can observe in Figure 2.The common practice [69] is to extract from the sensor response curves a smaller number of features, which allows reducing the dimensionality of the problem. In the present studies, we used a similar list of the modelling features as reported in previous papers [53,54,70]. We can enumerate several groups of the features types used in our data analysis. The first group consists of the response curve’s basic characteristics: maximum, minimum, median, average, standard deviation, skewness, and kurtosis. The second group consists of the same basic statistical types but calculated for the response curve derivative, calculated after the original curve smoothing using the exponential moving average method. Another group of features consists of characteristic times, such as the times to reach 10%, 25%, 50%, and 90% of the sensor’s response range, and the time to reach the maximum/minimum of the curve derivative. The next group of features consists of the magnitudes of the sensor response at characteristic moments of the measurements, such as half of the adsorption phase, the moment when it reaches the maximum/minimum of the response derivative. Finally, the last group of features consists of the parameters of the curve using a third-order polynomial. The modelling features were extracted separately (i) from the whole curve, (ii) from the adsorption, and (iii) from the desorption parts of the response.

### 4.2. Principal Component Analysis

Principal component analysis is an unsupervised method of data space transformation to a new space represented by factors. The magnitude of a factor is related to the variability of the dataset captured by the given factor. The factors construct a coordinate system in which the first coordinate vector sets the axis for which the variance of the dataset points is maximal, then the second coordinate vector sets the axis perpendicular to the first one and contains the maximum of the remaining variance of the dataset, and so on. The PCA transformation can be intuitively interpreted as a rotation of the coordinate system. One of the applications of PCA analysis is the reduction of the dimensionality of the dataset, as it allows the maximum of the content of the data variability in each of the components and decides to discard the less critical ones. In our analysis, the PCA method was used for visualisation purposes only. This allowed us to examine the data distribution in less dimensional space and understand the patterns. Since the input variables are represented in various measurement units in the studies we used and have various ranges, the input data were standardised, which allowed us to treat them on an equal footing.

### 4.3. Classification Modelling

To build a machine learning classification model, we used the logistic regression method. The model’s performance was evaluated using an accuracy measure, defined as the proportion of correctly classified samples to all samples in the scored dataset. The accuracy was calculated using the cross-validation (CV) technique.

It may be interesting to elaborate in more detail on the CV procedure used in our analysis. As we already mentioned, the measurements were performed over 10 days, and every day of measurement, each sample on a Petri dish was measured twice. In our opinion, that should be considered in the proper splitting of the experimental data to the training, validation, and testing datasets. Data collected from measurements of the same sample should not be used in both operations: model building and testing. In addition, as we noticed last time [70], the measurements performed during one day were pretty similar, and far more pronounced differences could be observed when we compared data from various days. That could be explained by varying measurements conditions, such as background odours, humidity, and temperature variations. An advantage of such a group splitting method is that it guarantees stratification of data and keeps the same proportion for each category of samples in a natural way. We implemented the CV loop so that data collected during two randomly selected days of measurements were kept apart as the testing dataset, and these data were used for model building or champion model selection, but only for calculations of the final performance statistics. The CV loop was used for 30 repetitions.

It is essential to notice that, in the dataset we prepared, the number of modelling features is quite large, and to avoid overfitting, we performed a variable selection procedure using the recursive variable selection method [70]. This task was performed inside the main CV loop, in another loop, and repeated 10 times. The partitions of the dataset, which were made in the main CV loop, were used again, one for model training and the second to evaluate its performance to select the best performing model (validation dataset). That means that the training dataset used in each step for fitting the logistic regression parameters contained only 60% of the original data. Additionally, for the training dataset, data standardisation was performed. The validation dataset was used for the choice of best features during the forward selection procedure. We limited this procedure to a choice of no more than 20 features. After the selection procedure for forward features, the performances of models with various numbers of features were compared, using statistics calculated from testing run on the validation dataset. The number of modelling features with which we achieved the best performing accuracy was found. We decided to implement this model training and selection procedure because it avoids leakage of information from the training/validation datasets to the testing dataset, and the final evaluation of the classification performance is performed on entirely independent data.

## 5. Results and Discussion

### 5.1. Principal Component Analysis

**Figure 4:**
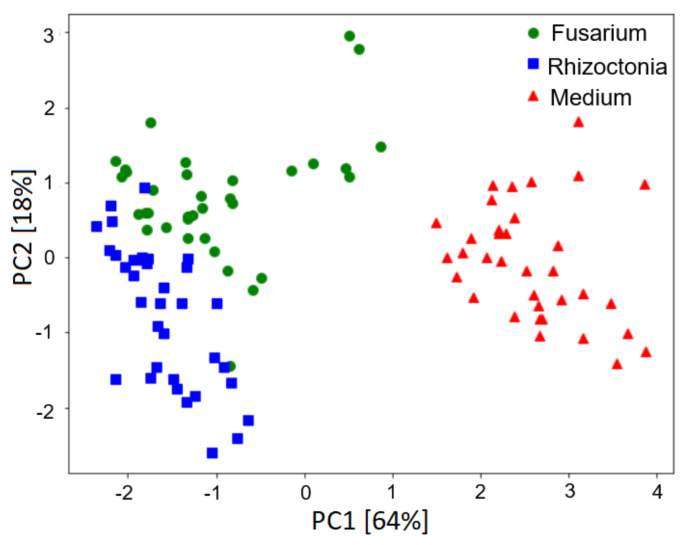
Distribution of measured samples based on PCA transformation of the modelling features extracted from sensor response curves. Data were obtained from the PW6 e-nose, all TGS 2603 sensors; six features selected by the classification model were transformed. The sample types are represented with different colours and symbols. The percentages of variability accounted for by the PC are indicated on the axes’ labels.

In Figure 4 we present results of the principal component analysis of the measurement data. As we described in the previous section, the input of the PCA was the modelling features extracted from the sensors’ response curves. The machine learning classification models selected the features that allowed for differentiation between the sample types. The PCA analysis was performed only to reduce the problem’s dimensionality, which helped with data visualisation.

### 5.2. Performances of the Classification Models

As one can observe in the following figure, there is a clear distinction between samples infected by fungi and non-infected samples containing only the growth medium. Additionally, we can notice that the separation between medium only and *Rhizoctonia* samples is larger than the separation between medium only and *Fusarium*. This may indicate that the classification models detecting *Rhizoctonia* perform better than the classification models differentiating between medium and *Fusarium* samples. In addition, we can observe that differentiation between the two studied species of fungi *F. oxysporum* and *R. solani* may be more challenging, as the data distributions for these samples are not totally separated and have a small overlapping region. As we present in the next section, such intuitive observations about the classification performances are confirmed by the accuracy measures based on the PCA visualisation.

**Figure 5:**
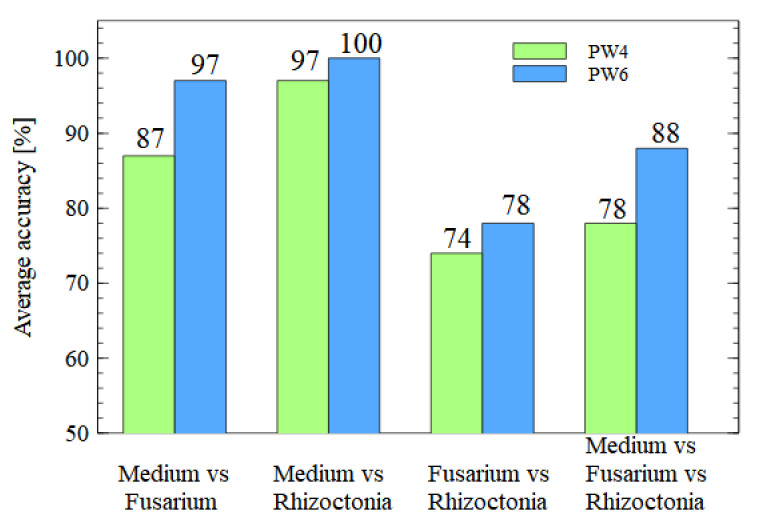
Comparison of the accuracy of the classification performance using data collected by the PW4 and PW6 electronic noses. Classification for binary or multinomial target models, as indicated on the x axis.

The first result of machine learning classification modelling that we would like to present compares the model’s performance using data collected by the PW4 and PW6 electronic noses. In Figure 5 we compare the accuracy of binary and multinomial target models for all combinations of sample types. The models were built using data collected by all sensors in electronic noses, which are listed in Table 1 and Table 2. As one can notice, an improvement in accuracy was achieved in all cases.

**Figure 6:**
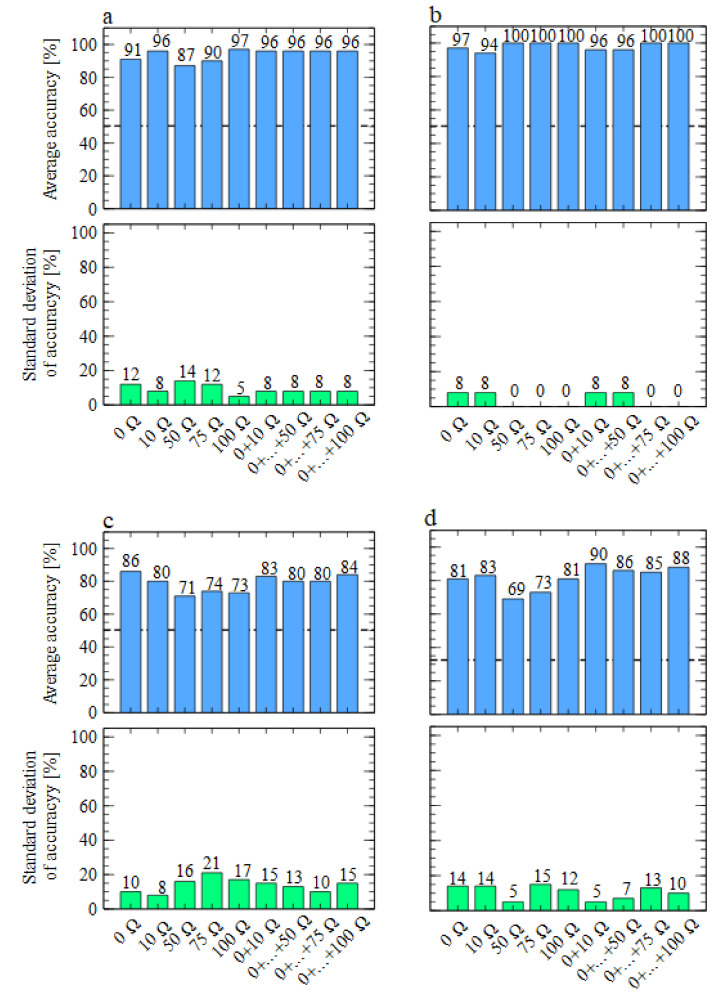
Performances of classification models, using data collected by the TGS 2603-type sensors with various heater voltages. Average accuracy and standard deviation of model accuracy were calculated in a cross-validation loop, as described in the main text. Binary or multinomial target classification is presented in subfigures. (**a**) Medium versus *Fusarium*. (**b**) Medium versus *Rhizoctonia*. (**c**) *Fusarium* versus *Rhizoctonia*. (**d**) Medium versus *Fusarium* versus *Rhizoctonia*. The bars represent various models built using a single sensor’s data or multiple sensors’ data, and the sensors are labelled by the resistances attached to their heaters. The horizontal line represents the null (random) model level.

For further analysis, we built more models to verify the performances of various cases, when data collected by just one of the sensors in the PW6 electronic nose were used for the model training, or when we used data collected from the sensor working at its heater voltage suggested by the manufacturer together with other sensors working at lower voltages (Table 2). We noticed that the data from TGS 2603 sensors provided better performances for the considered odours than the models built using TGS 2602 sensors’ data. For that reason, we would like to start our discussion from data presented in Figure 6, of the TGS 2603 sensors.

As one can notice, in most cases, we achieved better accuracy performance when the heater voltage was lower than the nominal one. In the case of differentiation between medium and *Fusarium* samples, the difference was in the order of six percent, which considering the number of data points, is equivalent to two more samples being correctly classified. The highest increase of the classification accuracy was observed for the multinomial classification when the classification of all categories was attempted, and the increase was in the order of nine percent, which is equivalent to about five samples.

**Figure 7:**
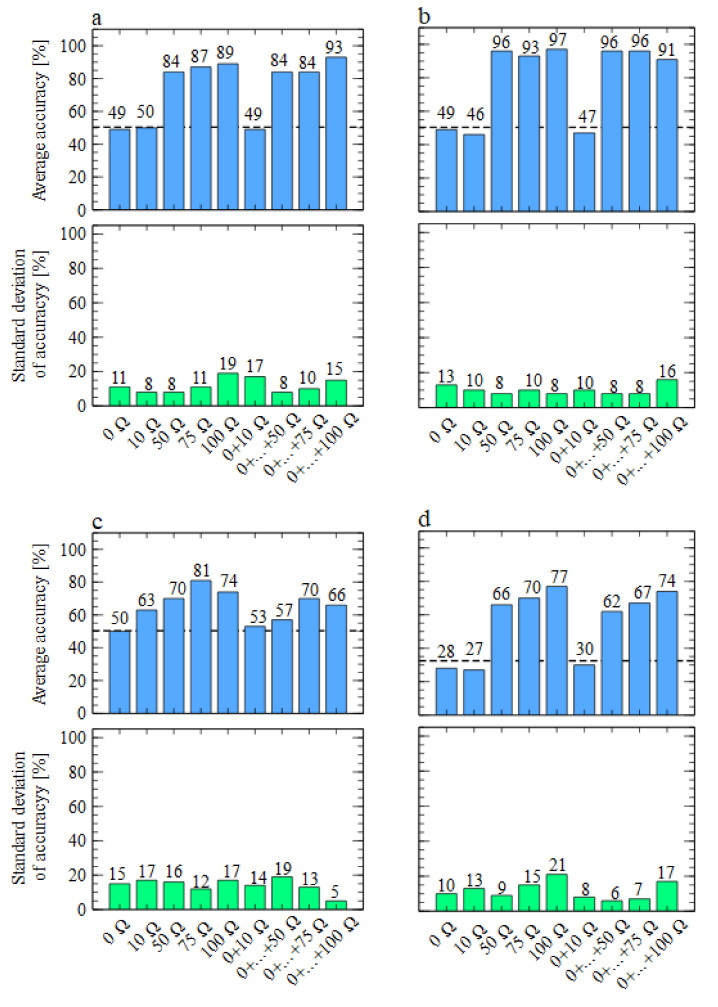
Performances of classification models, using data collected by the TGS 2602-type sensors with various heater voltages. Average accuracy and standard deviation of model accuracy were calculated in a cross-validation loop, as described in the main text. Binary or multinomial target classification is presented in subfigures. (**a**) Medium versus *Fusarium*. (**b**) Medium versus *Rhizoctonia*. (**c**) *Fusarium* versus *Rhizoctonia*. (**d**) Medium versus *Fusarium* versus *Rhizoctonia*. The bars represent various models built using a single sensor’s data or multiple sensors’ data, and the sensors are labelled by the resistances attached to their heaters. The horizontal line represents the null (random) model level.

In Figure 7 we present the results of model performance using the data collected by the TGS 2602-type sensors. As one can notice, in this case, we could not obtain good differentiation between the studied odours when sensors were working with the heater voltages recommended by the manufacturers. However, the models’ performances significantly improved for much lower voltages when resistors of 75 and 100 Ω were used. When we refer to the data presented in Table 2, we can notice that these resistors performed ideally when the sensors’ heater voltages were at most half of the nominal ones.

One final note is worth mentioning. As we discussed in the previous subsection, the distribution of data presented in Figure 4 could indicate perfect separation between clear medium and infected samples. Additionally, the separation between both categories of infected samples seemed to be potentially better than the results reported in Figure 5, Figure 6 and Figure 7. It can be recalled that the PCA transformation was based on the whole collected dataset, without separation of training and testing data. At the same time, the classification model’s performance was estimated using the training subsets of data not used in the model building process, and this was repeated for several splits in the cross-validation loop.

## 6. Summary

Electronic noses have been proposed as a rapid and non-invasive diagnostic tool in many industries and agriculture applications. In our previous papers [53,54] we demonstrated successful applications of these noses in differentiating between two species of pathogenic oomycetes, *Phytophthora* and *Pythium*. In the current paper, we reported further development of the constructed low-cost electronic nose device with an application to pathogenic fungal differentiation (*Fusarium oxysporum* and *Rhizoctonia solani*). Fungi of the genus *Fusarium* and *Rhizoctonia* are considered some of the most pathogenic and phytotoxic microorganisms in the world. They are plant pathogens with vast host ranges—worldwide, in fact. They are the most abundant pathogens in forest nurseries in Poland, where they cause damping-off, the disease that leads to the decay of germinating seeds and young seedlings.

The first low-cost electronic nose created by our team (PW4) [53,54] consisted of six Figaro Inc. TGS series gas sensors. The main idea behind developing the new e-nose (PW6) was another approach to selecting the sensors included in the sensor array. In the PW4 e-nose, we applied a broad range of TGS series sensors reacting to a broad range of chemical components. In the PW6 e-nose, we used only two sensors, which, in the preliminary measurements, provided data that allowed us to build the best performing classification models. This selection was made after experiments performed on oomycete odours, and we assumed that although they are technically not fungi, their odours should be similar to fungal ones; thus, this selection might be still valid. In the PW6 nose, we tested TGS 2602 and TGS 2603 gas sensors, for which various magnitudes of heater voltages were applied.

In the data analysis part of the research, we applied a well-established methodology. In the first step, the responses of the sensors to the presence of odours were transformed, and a set of features describing the shapes of the response curves were applied. Then the classification models using the logistic regression method were trained, and in this process, the variable selection procedure was applied. The accuracies of classification models were estimated using the cross-validation method.

The results demonstrated the possibility of differentiating between the studied samples using the data collected by the low-cost electronic nose devices. We also demonstrated an improvement of classification performance when data of the second proposed device were used.

## Figures and Tables

**Table 1:** A list of sensor models used in e-nose devices and the odours and gases targeted.

Sensor Model	Target Detection
*In both constructions (PW4 and PW6)*	
TGS 2602	Has high sensitivity to low concentrations of odorous gases such as ammonia and H${}_{2}$S generated from waste materials in office and home environments. The sensor also has a high sensitivity to low concentrations of VOCs such as toluene emitted from wood finishing and construction products. [55]
TGS 2603	Has high sensitivity to low concentrations of odorous gases such as amine-series and sulphurous odours generated from waste materials or spoiled foods such as fish. [56]
*Only in the first construction (PW4)*	
TGS 2600	Has a high sensitivity to low concentrations of gaseous air contaminants such as hydrogen and carbon monoxide, which exist in cigarette smoke. The sensor can detect hydrogen at a level of several ppm. [57]
TGS 2610	Uses filter material in its housing, eliminating the influence of interference gases such as alcohol, resulting in a highly selective response to LP gas. [58]
TGS 2611	Uses filter material in its housing which eliminates the influence of interference gases such as alcohol, resulting in a highly selective response to methane gas. [59]
TGS 2620	Has high sensitivity to organic solvents and other volatile vapours’ vapours, making it suitable for organic vapour detectors/alarms. [60]

**Table 2:** Resistors connected to each sensor’s heater supply circuit and the electric voltage applied to each sensor’s heater, in the PW6 electronic nose.

Resistor	TGS 2602	TGS 2603
0 Ω	5.0 V	5.0 V
10 Ω	4.5 V	4.6 V
50 Ω	3.0 V	3.2 V
75 Ω	2.4 V	2.6 V
100 Ω	2.0 V	2.3 V

**Table 3:** Power consumption of sensors’ heaters and other electronic components used in PW4 and PW6 electronic noses.

Component	Power Consumption
TGS 2600	210 mW
TGS 2602	280 mW
TGS 2603	240 mW
TGS 2610	280 mW
TGS 2611	280 mW
TGS 2600	210 mW
Other	250 mW
Total PW4	1750 mW
Total PW6	2850 mW

## Data Availability

The data presented in this study are available from the corresponding author.
